# The feasibility and usability of mixed reality teaching in a hospital setting based on self-reported perceptions of medical students

**DOI:** 10.1186/s12909-024-05591-z

**Published:** 2024-06-27

**Authors:** Michael Johnston, Megan O’Mahony, Niall O’Brien, Murray Connolly, Gabriella Iohom, Mohsin Kamal, Ahmed Shehata, George Shorten

**Affiliations:** 1 Peripheral Nerve Block Fellow, Cork University Hospital, Cork, Ireland; 2 Medical Student, University College Cork, Cork, Ireland; 3 Department of Anaesthesia, University College Cork, Cork, Ireland; 4 Coombe Women’s and Infants University Hospital, Dublin, Ireland; 5 Anaesthesiologist Cork University Hospital, University College Cork, Cork, Ireland

**Keywords:** Mixed reality, Augmented reality, Usability, Feasibility, Teaching, Undergraduate, Medical students, Microsoft HoloLens 2, Technology enhanced learning

## Abstract

**Background:**

Clinical teaching during encounters with real patients lies at the heart of medical education. Mixed reality (MR) using a Microsoft HoloLens 2 (HL2) offers the potential to address several challenges: including enabling remote learning; decreasing infection control risks; facilitating greater access to medical specialties; and enhancing learning by vertical integration of basic principles to clinical application. We aimed to assess the feasibility and usability of MR using the HL2 for teaching in a busy, tertiary referral university hospital.

**Methods:**

This prospective observational study examined the use of the HL2 to facilitate a live two-way broadcast of a clinician-patient encounter, to remotely situated third and fourth year medical students. System Usability Scale (SUS) Scores were elicited from participating medical students, clinician, and technician. Feedback was also elicited from participating patients. A modified Evaluation of Technology-Enhanced Learning Materials: Learner Perceptions Questionnaire (mETELM) was completed by medical students and patients.

**Results:**

This was a mixed methods prospective, observational study, undertaken in the Day of Surgery Assessment Unit. Forty-seven medical students participated. The mean SUS score for medical students was 71.4 (SD 15.4), clinician (SUS = 75) and technician (SUS = 70) indicating good usability. The mETELM Questionnaire using a 7-point Likert Scale demonstrated MR was perceived to be more beneficial than a PowerPoint presentation (Median = 7, Range 6–7). Opinion amongst the student cohort was divided as to whether the MR tutorial was as beneficial for learning as a live patient encounter would have been (Median = 5, Range 3–6). Students were positive about the prospect of incorporating of MR in future tutorials (Median = 7, Range 5–7). The patients’ mETELM results indicate the HL2 did not affect communication with the clinician (Median = 7, Range 7–7). The MR tutorial was preferred to a format based on small group teaching at the bedside (Median = 6, Range 4–7).

**Conclusions:**

Our study findings indicate that MR teaching using the HL2 demonstrates good usability characteristics for providing education to medical students at least in a clinical setting and under conditions similar to those of our study. Also, it is feasible to deliver to remotely located students, although certain practical constraints apply including Wi-Fi and audio quality.

## Background

*“He who studies medicine without books sails an uncharted sea, but he who studies medicine without patients does not go to sea at all.” William Osler *[1]*.* The value placed on medical teaching which directly involves patients is widely recognised by teachers and students. The value is attributed to the role it plays in situating learning in the students’ future workplace and to the opportunity to develop understanding of the relevance of basic science to clinical care (vertical integration) [2]. However, in many undergraduate medical programmes, the amount of time spent in teaching “at the bedside” has declined [2]. Reasons include the more rapid turnover of patients in hospital and improved forms of diagnostic tests.

In the light of the Covid 19 pandemic, the infection control risk of bringing students to the bedside has become a significant consideration, alongside the additional strain placed on the precious resource of personal protective equipment. Other considerations include the increasing requirement to provide remote learning and the environmental and wellbeing benefits of reducing the number of journeys medical students are required to undertake to attend clinical teaching. The centralisation of medical services has also led to medical students not having uniform exposure to all specialities. Clinical teaching with real patients in clinical tutorials lies at the heart of health professional education [3]. It provides students with opportunities to practice their history taking; physical examination; and communication skills [4]. In the discipline of anaesthesia, clinical judgement during preoperative assessment is critically important to minimise risks of rare events such as anaphylaxis [5] or difficulty in airway management [6].

Given the advances in technology-enhanced learning, the opportunity exists to support medical students in accessing and learning from clinical encounters by integrating selected artefacts to build and extend mental models from ‘bench-to-bedside’.

Mixed reality (MR) has been utilised in a diverse range of fields, including healthcare. There is now a body of work specifically looking at the applications of the Microsoft HoloLens 2 (HL2), a head-mounted display, MR Device within healthcare education.

The HL2 has been used intraoperatively to displaying complex holographic three-dimensional models based on patients’ pre-operative Computerised Tomography scans and aid surgical navigation [7]. A study by Bala et al. demonstrated the ability to use the HL2 to deliver a remote access MR ward round, in a real clinical setting and that this was found to be educationally effective and feasible [8]. Mill et al. also demonstrated live streaming of ward rounds using the HL2 and found the patient and student experience to be good but highlighted issues such as background noise, steep learning curves and Wi-Fi connectivity as barriers to delivery [9]. Wolf et al. utilised the HL2 to teach medical students the highly technical skill of extracorporeal membrane oxygenation cannulation; the MR group performed significantly fewer errors when compared with the conventional teaching group [10].

MR refers to a rendered experience blending the physical and digital world. The term mixed reality was first coined in a 1994 paper and defined as *merging of real and virtual worlds somewhere along the virtuality continuum which connects completely real environments to completely virtual ones’ *[11]*.* The blending of real and digital worlds allows users to interact and manipulate with real and virtual elements concurrently.

MR offers a potential solution to a number of challenges in medical education including providing students with meaningful access to patients, across different clinical specialties; infection control issues; and the provision of remote learning. Furthermore, it may serve as an effective means of supporting students in applying previously learned principles to the care of a specific patient who is present.

MR allows for the inclusion of digital artefacts into a real environment. This may offer potential to improve student learning by displaying three dimensional models; overlaying digital artefacts over live patients; utilising gestures and interactive features to highlight information to the learner. Combining audio-visual information may also reduce the cognitive load burden increasing learning efficacy [12].

Importantly, a MR supported clinical encounter may be used to enable students to associate their learning of basic scientific principles (for instance in anatomy or physiology) to relevant clinical applications; this could provide a valuable addition to teaching approaches integrating basic science knowledge and understanding with specific clinical challenges [13, 14].

The HL2 allows a live clinical encounter can be streamed to remote users. The HL2 is head-mounted device with a built-in camera which shows exactly what the tutor can see but with the addition of mixed reality allowing digital artefacts to be placed into the real environment. This stream of audio-visual data from the HL2 can be streamed to students situated remotely either on a single or to multiple devices. Bidirectional sound allows for the students, clinician, and patient to communicate in a manner similar to conventional bedside teaching. There is no upper limit on the number of students who could join the scenario remotely, unlike with conventional bedside teaching where only small groups are realistic. MR teaching may also uniform access and exposure to all specialities for medical students.

Given the inevitable challenges of introducing a new technology into the already complex healthcare environment we set out to examine the usability and feasibility of using the HL2 for teaching medical students. We examined this question using a prospective, observational, mixed methods study design. The tutorials were designed around clinical encounters with real patients during their perioperative pathway, and within a busy tertiary referral hospital.

## Methodology

The study was approved by the Clinical Research Ethics Committee of the Cork Teaching Hospitals. All patient and student participants provided written informed consent prior to participating in the study. All participants were aged over 18 years and deemed to have capacity to consent prior to enrolment in the study.

### Student participants

This study examined the use of the HL2 to facilitate a live two-way broadcast of a clinician patient encounter, utilising MR features of the HL2 device, which was streamed to Fourth Year Direct Entry and Third Year Graduate Entry University College Cork medical students. Medical students were invited to participate in the study which took place during their one-week placement in anaesthesia and intensive care medicine. Those invited to participate constituted a convenience sample as they were members of eight student groups (group size 3–10 students) scheduled to undertake this attachment from January 13th to March 31st 2023. The study took place in Cork University Hospital, a busy tertiary referral teaching hospital. All patients who participated were located on the Day of Surgery Assessment (DOSA) Unit having had their day case surgery completed. Background data was collected on medical students including age and previous degree level qualifications.

### Technical set-up

The tutorials took place on a once weekly basis from January to March 2023 (*n* = 8). All tutorials were delivered by a clinician investigator (MJ) and all sessions were facilitated by another investigator (NB). NB had significant prior experience with MR including specific experience with the HL2 Device.

NB facilitated the audio and visual connection between the clinical encounter and the tutorial room. MJ underwent informal training of how to use the Microsoft HoloLens 2 which took approximately 8 h to gain a working knowledge and an appreciation of the hand gestures required for smooth operation (comprising 4 h Microsoft remote assistance).

MJ wore the HL2 as a head mounted device and was face to face with the patient. The software package Microsoft Remote Assist alongside Microsoft Teams was used to enable a live two-way broadcast to the remote environment where the medical students were situated. The Cork University Hospital institutional Wi-Fi was used to transmit the broadcast. This provided a secure encrypted password protected network.

**Fig. 1:**
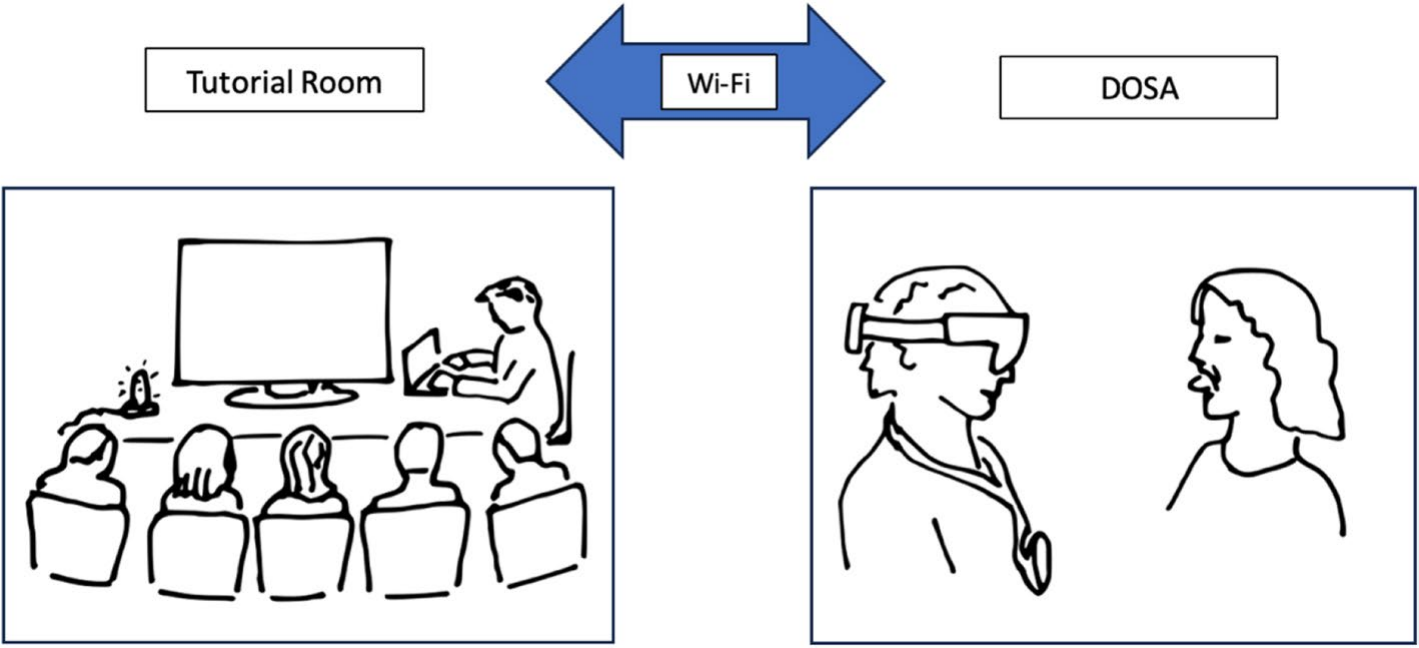
Physical set up of MR tutorial. Technician located with medical students in tutorial room. Clinician wearing HL2 with patient in Day of Surgery Admission Unit (DOSA)

Medical students were situated remotely in a tutorial room with the facilitator NB they were able to communicate directly with the clinician via a microphone and audio was provided via an external speaker. The clinician was able to communicate with the students via an external microphone on the HL2 Device and audio was provided by Headphones attached to HL2. The students were unable to communicate directly with the patient, but the clinician could act as a conduit for any questions (see Fig. 1).

Hardware requirements were as follows:

- Microsoft HoloLens 2
- Saramonic External Microphone (HoloLens 2)
- Apple 3.5mm Headphones (HoloLens 2)
- BeoPlay A1 Portable Bluetooth Speaker (Tutorial Room)
- Rode Compact Directional Microphone (Tutorial Room)
- Laptop capable of running Microsoft Teams
- HDMI cable
- HD Monitor

### Tutorial structure

At completion of their one-week anaesthesia and intensive care module the medical students attended for a mandatory three-hour tutorial comprising three sections; clinical case presentation; airway assessment; and 20 multiple choice questions on anaesthesia. Following the tutorial, the students had a 20-min break and then the students who had chosen to participate underwent the MR clinical airway tutorial. All MR tutorials were delivered by the same clinician (MJ) and the same technical facilitator (NB).

**Fig. 2:**
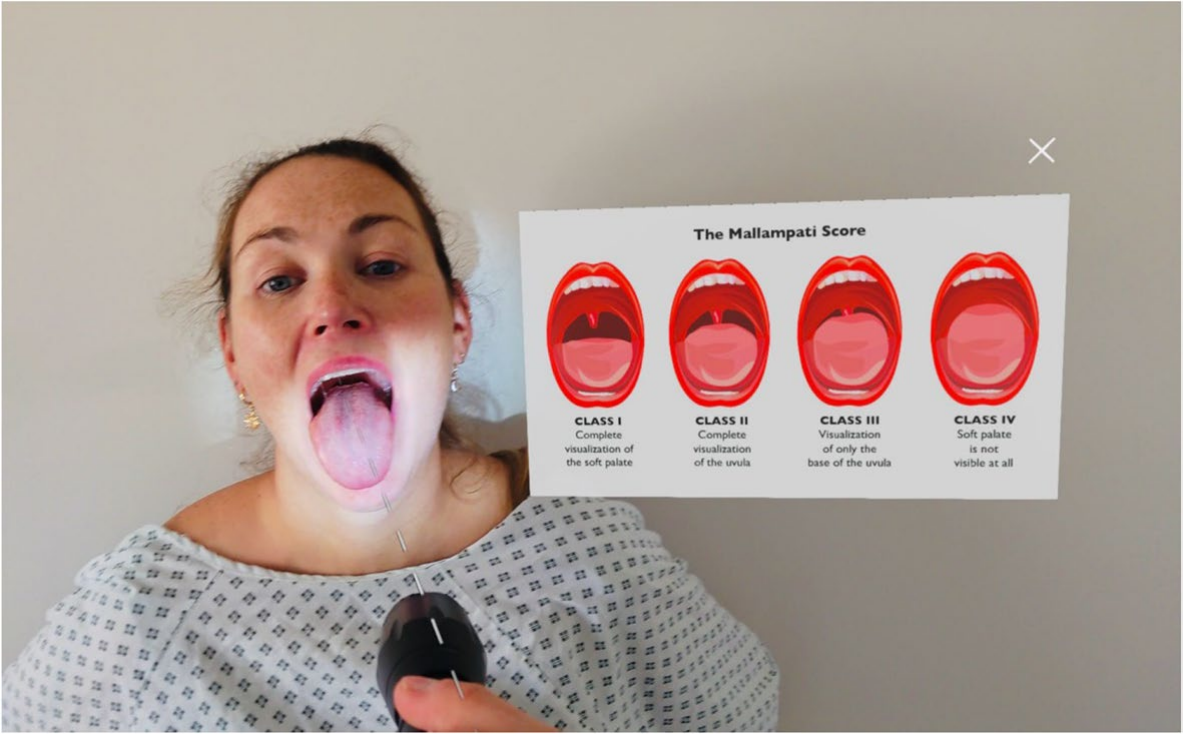
Artefact demonstrating Mallampati score alongside patient

The MR tutorial involved performing a basic airway assessment of a real patient and during this process the clinician was able to populate the shared view with artefacts to illustrate key teaching points such as the Mallampati score and mandibular protrusion test (see Fig. 2). A picture of a patient with several features of a difficult airway was then displayed and the students were asked to highlight concerning features. The second section of the tutorial focussed on clinical anatomy, a three-dimensional rendering of the upper airway was displayed, which could be rotated; zoomed in to; and highlighted using hand gestures within the HoloLens software. Collectively the students were asked to label the anatomical features and MJ further questioned the students on the clinical relevance of the anatomy.

### Cost

The following purchases were required to facilitate the tutorials. Microsoft HoloLens 2 device (€3500), Saramonic external microphone for HoloLens 2 (€88), headphones for HoloLens 2 (€10), portable BeoPlay speaker for tutorial room (€250), Rode microphone for tutorial room (€57), annual licence cost for HoloLens (€275) *n* = 2. In addition, NB provided his personal laptop, HDMI cable, and the tutorial room was equipped with a 36-inch, high-definition monitor to mirror the laptop screen onto.

### Assessment

The System Usability Scale (SUS) was employed to assess the usability of the MR environment for teaching. SUS were completed by all 3 of the relevant parties: medical students, teaching clinician, and technician. SUS forms were completed immediately after the tutorial.

A modified Evaluation of Technology-Enhanced Learning Materials: Learner Perceptions Questionnaire (mETELM) was completed by the medical students and patients, which used a 7-point Likert scale. The questionnaire included questions on previous experience with mixed or virtual reality; feasibility data relating to visual clarity and three-way audio broadcast; and data about how the MR tutorial compared with both a conventional PowerPoint presentation and in-person bedside teaching. There was also an area for free text feedback.

Although learning efficacy attributable to the HL2 use was not formally or comprehensively assessed, we examined academic performance (based on the end of year Objective Structured Clinical Examinations results in the airway examination station) for students who attended the MR tutorial compared with those who just underwent the conventional teaching.

## Results

This was a mixed methods prospective observational study, which took place in a busy tertiary referral hospital in the Day of Surgery Assessment Unit involving real patients on their perioperative journey.

Eight clinical tutorials involving 8 separate patients took place between 13/1/23 and 31/3/23. Of a total of 53 medical students who were eligible to participate in this study (as scheduled to attend MR tutorial) 47 elected to participate having provided written informed consent. The 47 medical students comprised of 21 males and 26 females. The participants comprised 18 graduate entry third medical students (mean age = 28.5 years, SD = 3.78) and 29 Direct entry fourth year medical students (mean age = 22.3 years, SD = 0.97).

All 47 medical students fully completed the SUS and the mETELM Questionnaire. The Clinician and technician also completed the SUS.

Of the 8 patients who participated (having provided written informed consent), 7 completed the mETELM questionnaire. 1 patient did not complete the mETELM due to feeling nauseous.

### Usability

**Fig. 3:**
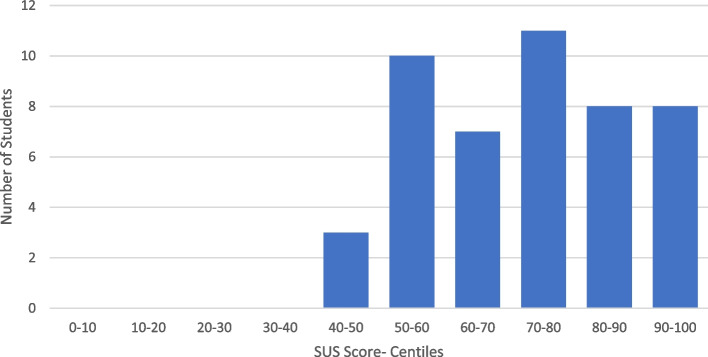
SUS Score- Medical Students

Overall, a mean score of 71.4 (SD = 15.4) on the SUS was elicited from the 47 participating medical students (See Fig. 3). A SUS score of 68 is considered to demonstrate satisfactory usability, with a score of 68- 80.3 considered good usability and > 80.3 considered excellent usability [15]. Therefore, we can state that, in the context of our study, the medical students found the HL2 to have good usability characteristics.

The clinician/teacher (MJ) and education technician (NB) also completed the SUS (score = 75 and 70 respectively). The clinician/teacher and technician were generally positive about use of the device. The clinician teacher found the HL2 to be comfortable to wear, to provide an unrestricted field of view and have good battery life. However, he found that the process of opening up the three-dimensional airway model to be time consuming, as it involved opening Microsoft edge web browser and then opening and expanding the model, (taking approximately 30 s).

### Student and patient mETELM questionnaires

**Fig. 4:**
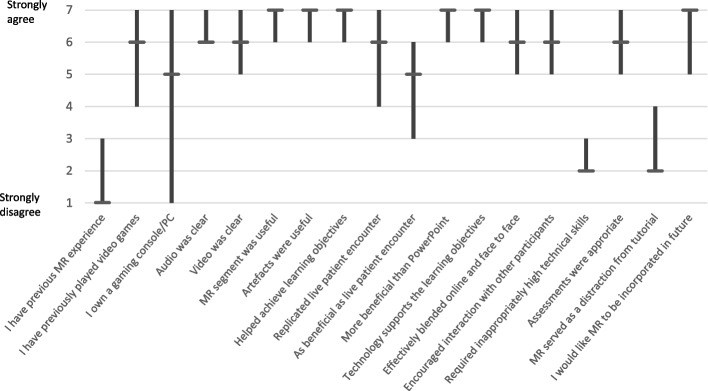
Students mETELM Questionnaire responses. Median and Interquartile Range displayed

The students mETELM Questionnaire responses are displayed in Fig. 4. There was limited previous experience with MR, but significant experience with gaming consoles and computers. Audio and video quality was felt to be clear. The MR tutorial was felt to be universally more beneficial than a PowerPoint presentation and replicate a live patient encounter. The results were borderline for if the MR tutorial was as beneficial as a live patient encounter. MR seemed to support learning, and there was a positive response to further MR teaching being incorporated into future tutorials. It was felt that the tutorial did not require inappropriately high technical skills, and that MR was not a distraction from the content of the tutorial.

**Fig. 5:**
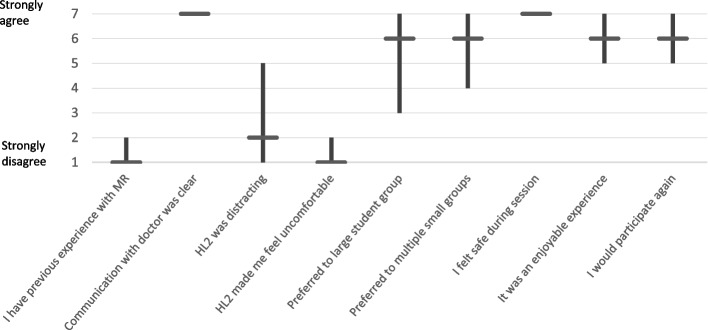
Patient mETELM Questionnaire responses. Median and interquartile range displayed feasibility

The patients mETELM Questionnaire responses (see Fig. 5) suggest the HL2 did not affect communication with the doctor but did form a distraction to some patients. The HL2 did not make patients feel uncomfortable and they felt safe during the sessions. Interestingly, patients collectively expressed a preference for the MR supported tutorial format over small or large student group tutorials at their bedside; most patients described the MR enabled tutorials as an enjoyable experience and indicated that they would favour participating in similar tutorials again.

Overall, we found that it was feasible to deliver teaching using the HL2 in a live clinical environment, but we encountered certain significant barriers. The institutional Wi-Fi was generally acceptable for providing adequate quality audio and visual streams, but on occasion connectivity dropped and was not resumed until the laptop and HL2 had both been restarted. Our occasional attempts to record a session would significantly reduce the quality of the connection, forcing us to restart both the HL2 and laptop. Despite this we always managed to run the MR tutorials but sometimes there would be delays of up to 15 min whilst we established a good quality connection. After 2 tutorials we improved the student’s ability to communicate with the clinician with the addition of an external microphone in the tutorial room.

Prior to delivery of the teaching the clinician had to learn how to use the HL2 device, a process which took around 8 h. The technician was already familiar with the HL2 and Microsoft Teams and Remote Assist. There were significant costs in terms of hardware, software, and licensing to run MR tutorials with the HL2.

### Additional findings

Representative quotes from students’ free text are on mETELM questionnaire are detailed below in Table 1.

**Table 1:** Representative Quotes from free text area mETELM Questionnaire

Negatives	Positives
Issue with Wi-Fi and video dropping out	So much better than another PowerPoint presentation
Would be great to overlay three-dimensional airway model onto the patient	Very helpful, MR really helped to clarify topics
Improve sound quality, tutor couldn’t hear us very well	Images very useful and three-dimensional model was great
Bigger monitor and higher resolution would help	Great to have textbook image side by side with real patient
Set up could be quicker	Point of view presentation much more helpful than conventional teaching
Angle of camera meant we couldn’t visualise patient’s Mallampati score	Great potential, I really want to see it incorporated into more of our tutorials
	Great to have everything in one place, much better than reading a book and then just being expected to know everything

The medical students completed an end of year OSCE on airway examination. The OSCE was 50% related to what we taught in the HL2 tutorial and 50% related to other aspects of airway management. The interval between the HL2 tutorial and the OSCE varied significantly between students from 6 weeks to 4 months. The airway OSCE was scored out of 50. The scores for students who completed the HL2 tutorial (*n* = 47) and those who had conventional teaching only (*n* = 139) were similar (HL2 teaching- mean 45.1, SD 4.9, versus conventional teaching- mean 44.1, SD 7.0). The unpaired student t-test two tailed value was 0.58. No difference in learning efficacy was found between the HL2 group and those who underwent conventional teaching.

## Discussion

The most important finding of this study is that the HL2 demonstrates good usability characteristics for providing clinical education to medical students in a real clinical setting. Within the infrastructure of a busy teaching hospital and using institutional Wi-Fi, it is feasible to deliver bedside clinical teaching to a group of remotely located medical students. We propose that MR-enhanced teaching may allow for educational institutions to provide consistent and uniform access to clinical teaching.

We also found that students expressed great openness to and enthusiasm for this way of learning, both in their Questionnaire responses and at semi structured interview. This provides encouragement for future use of MR devices within the clinical space.

Our findings are broadly consistent with others which have demonstrated that that the teaching of medical students within a variety of clinical environments using MR showed good usability and feasibility characteristics and are generally acceptable to patients, students, and teachers [8, 9, 16–18]. Mill et al. highlighted some issues similar to those we experienced regarding Wi-Fi connectivity, and background noise disrupting sessions [9]. Our study results are also consistent with those of Levy et al. in that some patients preferred and felt more comfortable having a single clinician wearing a HL2 compared with having a group of healthcare professionals at the bedside [19].

A review article by McBain et al. examined the effect of using virtual, augmented and mixed reality to teach clinical anatomy to medical students using three dimensional digital models and our study showed globally similar positive findings with regards to usability and feasibility [20]. Other investigators have also noted (in the context of a clinical teaching during a pandemic) that the HL2 can facilitate ongoing delivery of clinical teaching with a lesser need for personal protective equipment use and increased safety for all [21].

Our study was relatively novel as it was implemented within a busy clinical environment with real patients on their perioperative pathway. The usability data we collected employed two different tools (SUS and mETELM) and rendered similarly positive results. We interpret that similarity as supporting the validity of our findings. Our usability dataset was elicited from the different perspectives of medical students, a clinician, patients, and a technician. (Only one patient did not complete the mETELM Questionnaire).

The ETELM instruments were designed originally by Cook et al. [22] using a combined inductive/ deductive approach and comprehensive literature review to ensure that relevant domains were captured (based on frameworks widely applied to mainstream education) and inclusion of specific items of relevance to health professionals’ education. The designers specify that the ETELM instrument is intended to be adapted to “context and situation-specific needs” [22]. We did not conduct within-study assessments of face, content, or test–retest validity of the mETELM. The risk that the information elicited using this instrument is invalid may be offset by the selection of an instrument specifically designed both for educational technology and for health professional learners.

All MR teaching was delivered by a single clinician which ensured uniformity of teaching style and approach. Three-way communication between patient, clinician and medical students allowed for communication in a similar style to in person bedside teaching. A different patient participated in each clinical tutorial leading to a diverse range of clinical findings and discussion points.

Utilising the HL2 technology to provide both a clinical tutorial involving a real patient and then progressing onto three-dimensional anatomy teaching demonstrated the capabilities of the device, utilising the basic audio-visual streaming features, with adjacent MR artefacts.

This was a relatively small study with a limited number (*n* = 47) of medical students participating. The study only focused on a single highly specific clinical domain. The study took place in one location (DOSA) in one hospital; therefore, our findings cannot necessarily be extrapolated to other hospital environments. Because the patients who participated were all elective patients on a day case surgical pathway, our findings cannot necessarily be extrapolated to patients of different backgrounds or differing degrees of acuity. We did not assess the feasibility of streaming the sessions to multiple personal devices.

Use of MR clinical teaching via a device like the HL2 is feasible and has positive usability characteristics. The feedback reported in this study indicates that MR offers potential for technology enhanced learning and specifically to improve vertical integration within an undergraduate medical curriculum.

There are many potential future applications of MR technology within the healthcare setting. These may include bedside teaching in more diverse environments, sharing three dimensional models during surgery, anatomy teaching including specific applications within regional anaesthesia.

One possibility to scale a MR enabled learning resource would be a library of MR teaching sessions which students would be able to access at their own discretion to allow for uniform access to clinical teaching and a potentially unlimited number of students to participate. This set up would be enhanced further by collecting engagement data from students and embedding pop quizzes into the tutorials. Allowing students to use their own devices to join sessions clearly offers practicality benefits to the students and environmental benefits but raises questions regarding data protection and maintaining confidentiality.

Although we do not provide here enough information to provide a needs analysis for scaling up or transferring the type of mixed reality teaching described to other institutions, our results indicate that faculty training, suitably chosen hardware and software, secure and reliable Wi-Fi will be pre-requisites.

The HL2 has a training curve associated with basic competence and an interesting area of research would be to ascertain how long it would take a group of clinicians to become competent to deliver a clinical tutorial using the device. With a larger group of trained clinicians, a more diverse range of subjects could be delivered using MR.

Our results point to the likelihood of employing MR to apply basic science principles to clinical practice (including diagnosis and management decisions). It will be important to examine whether this potential can be realised without distracting or overwhelming the learner. Further work on this question is warranted.

## Conclusions

The most important finding of this study is that MR supported teaching using the HL2 demonstrates good usability characteristics for providing clinical education to medical students at least in a clinical setting and under circumstances similar to those we describe here. Within the infrastructure of a busy teaching hospital and using institutional Wi-Fi it is feasible to deliver teaching related to clinical encounters to remotely located medical students, although certain practical constraints apply including the quality of Wi-Fi and audio connections.

## Data Availability

Any data used by the authors can be made available if requested. Please contact corresponding author Michael Johnston if required.

## Abbreviations

MR: Mixed reality
HL2: Microsoft HoloLens 2
mETELM: modified Evaluation of Technology-Enhanced Learning Materials: Learner Perceptions Questionnaire
SUS: System Usability Scale
DOSA: Day of Surgery Assessment Unit
UCC: University College Cork
